# Association between statin usage and mortality outcomes in aging U.S. cancer survivors: a nationwide cohort study

**DOI:** 10.1007/s40520-024-02851-2

**Published:** 2024-10-05

**Authors:** Shan Ding, Fengling Yang, Pan Lai, Weiwen Jiang, Minze Chen, Yijun Ge, Liting Zhou, Shaozhuang Chen, Jiaqi Zhang, Yanrong Ye

**Affiliations:** 1 Zhongshan Hospital (Xiamen), Fudan University, Xiamen, 361015 China; 2 Longyan First Affiliated Hospital of Fujian Medical University, Longyan, 364000 China; 3 Peking University First Hospital, Beijing, 100034 China; 4 Zhongshan Hospital, Fudan University, Shanghai, 200032 China; 5 Faculty of Health and Environmental Sciences, Auckland University of Technology, Auckland, 0627 New Zealand; 6 Shenzhen Campus of Sun Yat-Sen University, Shenzhen, 518107 China

**Keywords:** Cancer survivors, Mortality, Statins, NHANES, Aging

## Abstract

**Background:**

The population of Aging cancer survivors in the United States has surged to over 16.9 million. Research on the relationship between statin usage and post-cancer survival rates remains limited.

**Aims:**

This study aims to investigate the association between statin use and various causes of mortality among aging cancer survivors.

**Methods:**

We analyzed NHANES data from 1999 to 2018, Statin usage, both hydrophilic and lipophilic, was derived from NHANES prescription records. We utilized Cox proportional hazards models to associate statin utilization with mortality, differentiating causes of death according to statin type and patterns of use.

**Results:**

Within a cohort of 2,968 participants, statin usage was categorized into non-users (1,738), hydrophilic statin users (216), and lipophilic statin users (982). Compared to those who did not use statins, individuals prescribed hydrophilic statins did not show a reduced risk of all-cause mortality (adjusted hazard ratio [HR] 1.01; 95% confidence interval [CI] 0.72–1.41; *P* = 0.960), as outlined in Model 3. In contrast, the group receiving lipophilic statins exhibited a notable decrease in all-cause mortality risk (adjusted HR, 0.77; *P* = 0.003). Nonetheless, both hydrophilic and lipophilic statins were effective in diminishing the risk associated with cancer from its onset until death, with hydrophilic statins showing a greater level of efficacy.

**Discussion:**

The potential of statins to reduce cancer-related mortality may provide avenues for targeted clinical interventions and management strategies.

**Conclusions:**

Our study reveals that the use of lipophilic statins is significantly associated with lower all-cause and cancer-cause mortality risks among aging cancer survivors.

**Supplementary Information:**

The online version contains supplementary material available at 10.1007/s40520-024-02851-2.

## Introduction

As advancements in cancer therapies continue to flourish, the population of cancer survivors is expanding at a remarkable rate, with over 16.9 million individuals in the United States who have conquered the disease [1]. Within this extensive community, as we embrace the era of aging, cancer survivors are progressively transitioning into their elderly phase. However, this group faces the ongoing and delayed effects of various cancers and their treatments, which regrettably might reduce life expectancy [2]. Consequently, it is imperative to accelerate the development of viable strategies that these elder survivors can adopt to enhance their long-term health outcomes.

Statins, are renowned for their ability to lower blood cholesterol levels and serve as preventative and therapeutic agents in the realm of cardiovascular disease with pronounced efficacy and minimal side effects [3, 4]. Increased evidence suggests that the benefits of statins extend beyond their cardiovascular applications. They are associated with a diminished occurrence of neurological disorders [5], as well as a decreased mortality risk in the context of breast [6, 7], prostate [8], colorectal [9], and renal cell carcinomas [10]. Moreover, these compounds exhibit pleiotropic properties that span anti-inflammatory and anti-tumoral cell proliferation activities [11–13], potentially harbouring stand-alone anti-cancer effects [14]. Statins are classified into hydrophilic and lipophilic statins, which have different intracellular effects depending on their chemical structure. Lipophilic statins show a greater ability to penetrate the cell membrane and also have higher pro-apoptotic activity than hydrophilic statins [15, 16]. Due to this higher cytotoxic potential, lipophilic statins may be beneficial in cancer treatment [17]. There is a noticeable lack of research on the relationship between statin use and post-cancer survival rates, especially among older cancer survivors. Moreover, additional epidemiological studies are necessary to shed light on the synergistic effects and potential advantages of using different types of statins(hydrophilic and lipophilic) to improve survival following a cancer diagnosis.

In the present study, we used the 1999–2018 National Health and Nutrition Examination Survey (NHANES) data, to detect the relationships of statins use with the mortality of aging cancer survivors to investigate whether statin use may improve long-term health outcomes of aging cancer survivors.

## Methods

### Study population

The National Health and Nutrition Examination Survey (NHANES) is a series of independent, nationally representative cross-sectional surveys conducted by the National Center for Health Statistics. Detailed information on recruitment, procedures, population characteristics, and study design is available through the Centers for Disease Control and Prevention (https://www.cdc.gov/nchs/nhanes/index.htm). NHANES was approved by the National Center for Health Statistics Research Ethics Review Board, and all participants provided written informed consent.

**Fig. 1:**
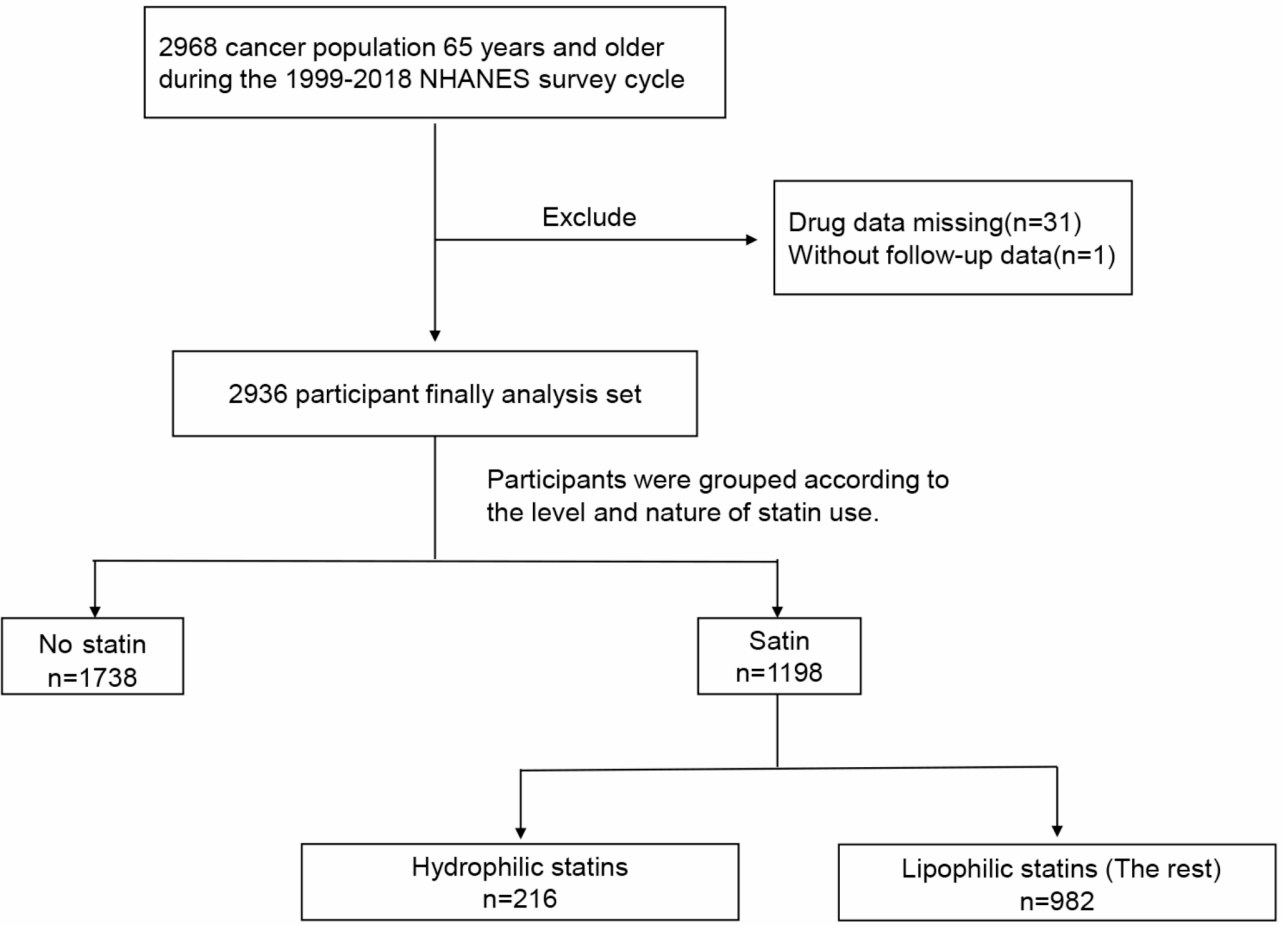
Flowchart

For our study, we utilized NHANES 1999–2018 data from cancer survivors aged 65 years or older. We excluded missing medication data population (*n* = 31), and missing follow-up information population (*n* = 1). Resulting in 2,936 adult subjects were included in the final analysis (Fig. 1)

### Exposure and study variables

In the NHANES database, participants reported drug use in the prescription drug section of the sample person questionnaire interview, providing categories of therapeutic drugs associated with each drug and ingredient. Additionally, the use of statins occurs after the diagnosis of cancer. Statin users were defined by identifying unique generic codes from the Multum Lexicon drug database [18]. We used the following prescription drugs as statins for this analysis/The prescription drugs considered as statins for this analysis were: lipophilic statins (simvastatin, fluvastatin, pitavastatin, lovastatin and atorvastatin) and hydrophilic statins (rosuvastatin and pravastatin). In our cohort, participants were categorized based on statin use and the use of different types of statins: (1) No statin group; (2) Statin group: (a) Hydrophilic statins; (b) Lipophilic statins (The rest).

### Diagnosis of cancer

Information regarding cancer diagnoses was gathered through face-to-face interviews.

These interviews included details on up to three types of cancer diagnoses and the respective ages at which each diagnosis occurred. Participants were asked, “Has a doctor or another health professional ever informed you of having cancer or any form of malignancy?” Those affirming this question were identified as cancer survivors and further queried about the specific type of cancer and their age at the time of initial diagnosis. The time elapsed since the first cancer diagnosis was determined by subtracting the age at the first diagnosis from the current age of the participant. eTable 1 provides a detailed breakdown of cancer survivors by type and sex, based on data from the NHANES 2007–2018.

### Defining variables of interest

Participants self-reported age, gender, race, and education. CVD was defined for self-reported coronary heart disease or congestive heart failure or angina or heart attack or stroke [19]. Dyslipidemia was defined for LDL > 130 mg/dL or HDL < 40 mg/dL, and was independent of the use of statins. Diabetes was defined by treatment of diabetes, self-reported history of diabetes, or glycosylated hemoglobin (HbA1c) ≥ 6.5% [20]. Hypertension was defined by using the 2017 ACC/AHA/ MS guidelines [21]. Participants were classified as smokers if they smoked ≥ 100 cigarettes during their lifetime [22].

### Determination of mortality outcomes

NHANES is associated with death certificate records in the National Death Index (NDI), which determines the outcome of a death by linkage to National Death Index (NDI) records [23]. Cause-specific deaths are determined according to the International Classification of Diseases, Tenth Revision (ICD-10) identified and National Center for Health Statistics (NCHS) classified cardiac diseases (054–064), malignant neoplasms (019–043), and all other causes (010) [24]. Mortality follow-up data as of December 31, 2019 for 1999–2018 NHANES participants.

### Statistical analysis

In accordance with the instructions for using NHANES data, we considered complex survey design factors. Their recommended weights were used for analysis, and continuous variables were expressed as mean ± standard deviation and statistically analyzed using one-way ANOVA, while for categorical variables counts and proportions (after weighting) were given, using card method tests [25].

To assess the incidence rate of all-cause mortality events across groups defined by varying levels of statin usage, we employed Kaplan-Meier survival analysis, with differences among the groups being assessed using log-rank tests. In our study, stratified Cox proportional hazard modeling was utilized to explore the relationship between endpoints and statin use. We classified causes of death into four categories: all-cause mortality, cardiovascular mortality, cancer-related mortality, and mortality from other causes. The data were categorized according to the use of statins and the use of different types of statins as follows: (1) No statin group; (2) Statin group: (a) Hydrophilic statins; (b) Lipophilic statins (The rest). Three Cox regression models were developed to examine the association between statin use and mortality in the cancer population: Model 1 (unadjusted); Model 2 adjusted for gender, age, and race/ethnicity; and Model 3 adjusted for gender, age, race/ethnicity, education level, BMI, smoking status, alcohol use, dyslipidemia, hypertension, diabetes, and CVD. To ascertain the reliability of the association between statin use and cancer survivors, we analyzed the risk of cancer diagnosis to death in relation to statin usage. Additionally, we conducted sensitivity analyses by excluding deaths within the first two years of observation, followed by a re-evaluation of the primary outcomes.

All analyses were performed using R 4.3.1 as well as a two-sided *P*-value < 0.05 indicating that all analyses were significant.

## Result

Among the cohort of 2,968 aging evaluated, 1,738 did not (utilize/use) statins, 216 were prescribed hydrophilic statins, and 982 were administered lipophilic statins, accounting for the remainder. The baseline characteristics of the participants, categorized by the extent and type of statin usage, are depicted in Table 1. When juxtaposed with the non-statin group, individuals in the statin group were exhibited a decreased proportion of male constituents (*P* < 0.001). Moreover, a higher prevalence of CVD, hypertension, and diabetes mellitus were observed in no statin group, yet a diminished frequency of dyslipidemia were noted. However, the LDL-C, HDL-C, and TC were markedly decreased in the statin group in contrast to their counterparts in the no statin group, with all noted differences reaching statistical significance (*P* < 0.001).

**Table 1:** Baseline characteristics of statin users and nonusers among aing cancer survivors—NHAMES 1999–2018

Variable	Total	No statin	Statin		*P*-value
			Hydrophilic statins	Lipophilic statins (The rest)	
**Age (year)**, **SD**	74.5 ± 0.2	74.5 ± 0.2	75.2 ± 0.4	74.2 ± 0.2	0.079
**Female**, **n (%)**	28.3(0.1)	27.7(0.2)	28.8(0.5)	29.1(0.2)	< 0.001
**Race**, **n (%)**					0.629
Mexican American	149(1.4)	95(1.4)	10(1.1)	44(1.5)	
Non-Hispanic Black	360(4.7)	210(4.7)	22(3.6)	128(4.8)	
Non-Hispanic White	2209(89.3)	1302(89.1)	168(91.6)	739(89.1)	
Other Hispanic	111(1.8)	72(2.1)	6(1.1)	33(1.4)	
Other Race	107(2.9)	59(2.7)	10(2.6)	38(3.3)	
**Education level**, **n (%)**					0.162
< 12	756(18.2)	461(19.4)	52(16.4)	243(16.6)	
12	1065(35.7)	644(35.8)	66(30.4)	355(36.7)	
> 12	1110(46.0)	629(44.8)	98(53.1)	383(46.7)	
**Smoking status**, **n (%)**	1646(55.2)	926(52.3)	130(56.4)	590(60.0)	0.014
**Alcohol using**, **n (%)**	2216(77.9)	1295(84.7)	162(82.1)	759(88.2)	0.066
**BMI (kg/m2)**, **SD**	28.3 ± 0.1	27.7 ± 0.2	28.8 ± 0.5	29.1 ± 0.2	< 0.001
**LDL-C (mg/dl)**, **SD**	110.7 ± 1.3	123.1 ± 1.6	94.1 ± 3.5	91.9 ± 1.8	< 0.001
**HDL-C (mg/dl)**, **SD**	55.8 ± 0.4	57.5 ± 0.6	52.7 ± 1.4	53.5 ± 0.7	< 0.001
**TC (mg/dl)**, **SD**	192.9 ± 1.1	207.9 ± 1.2	169.1 ± 2.7	171.9 ± 1.7	< 0.001
**TG (mg/dl)**, **SD**	150.9 ± 2.4	148.2 ± 2.8	155.2 ± 7.5	154.6 ± 4.1	0.294
**Family DM**, **n (%)**	1088(36.2)	610(34.3)	87(38.4)	391(39.0)	0.240
**Family CVD**, **n (%)**	356(13.3)	183(11.6)	37(17.9)	136(15.3)	0.024
**CVD**, **n (%)**	937(29.4)	423(22.9)	92(39.0)	422(38.7)	< 0.001
**Hypertension**, **n (%)**	2377(79.6)	1360(76.7)	186(86.2)	831(83.4)	0.005
**DM**, **n (%)**	891(27.6)	419(21.9)	80(37.2)	392(35.6)	< 0.001
**Dyslipidemia**, **n (%)**	747(25.0)	496(30.8)	51(25.0)	200(20.5)	< 0.001

*Abbreviations* BMI, Body mass index; LDL-C Low-density lipoprotein-C; HDL-C high-density lipoprotein cholesterol; TC, Total cholesterol; TG, triglyceride; CVD: cardiovascular disease; DM, diabetes mellitus

**Fig. 2:**
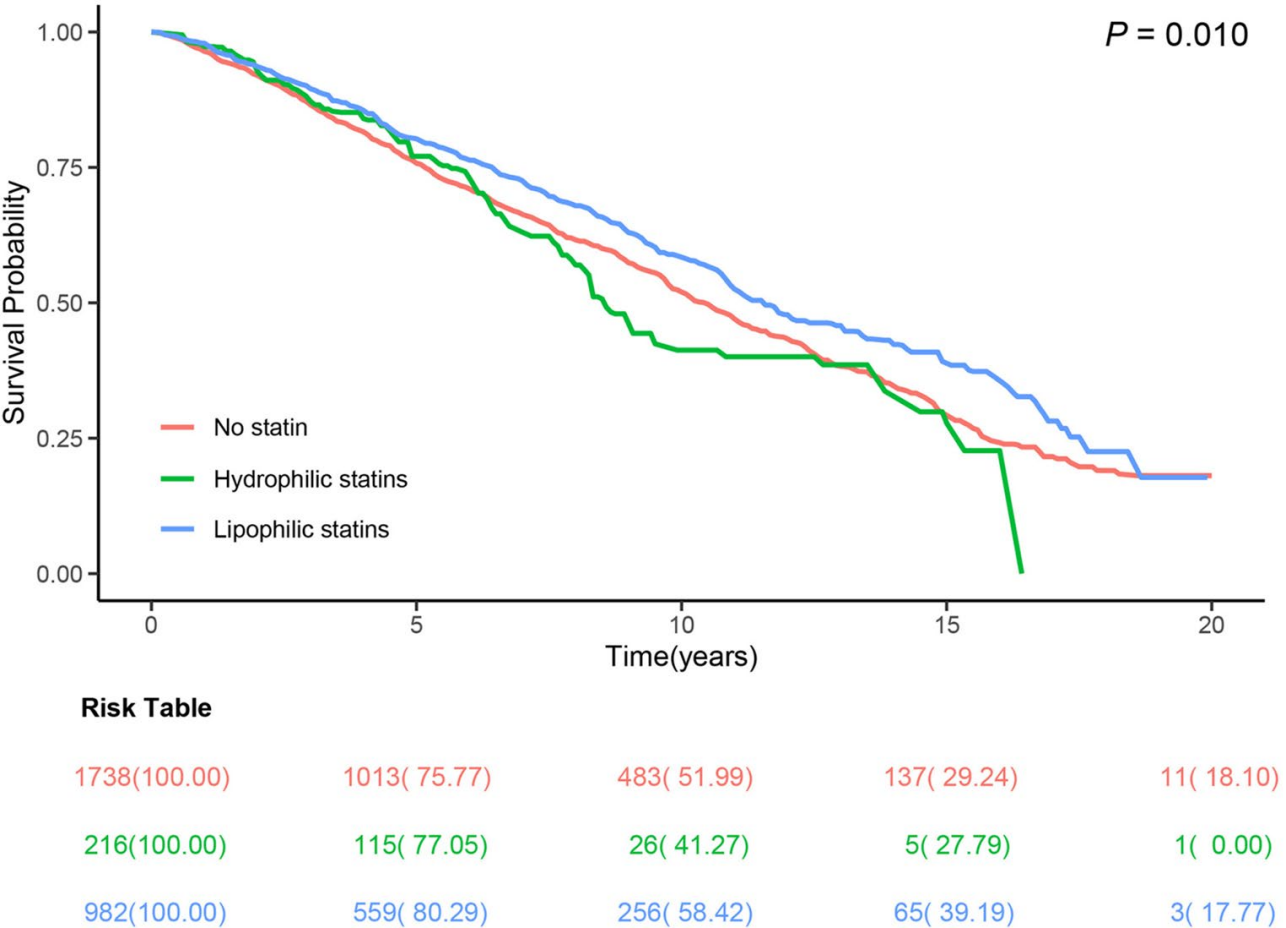
Kaplan–Meier survival analysis curves for all-cause mortality

The Fig. 2 illustrates survival probability over time for two statin users and non-user groups, showing significant differences in survival rates (*P* = 0.010). The risk table provides detailed counts of individuals at various time points, reflecting changes in survival across the groups. In contrast to nonuser group, the cohort prescribed hydrophilic statins did not exhibit a diminished risk in terms of all-cause mortality (adjusted hazard ratio [HR], 1.01; 95% confidence interval [CI], 0.72–1.41; *P* = 0.960), as delineated in Model 3 (Table 2). Conversely, a significant reduction in all-cause mortality risk was observed in the cohort administered lipophilic statins (adjusted HR, 0.77; *P* = 0.003). To elucidate the underlying causes of mortality in greater detail, we analyzed subsets of patients who succumbed to cancer-related fatalities, cardiovascular incidents, or other mortality etiologies. Notably, those administered lipophilic statins all demonstrated a significantly diminished cancer mortality risk, cardiovascular mortality risk, and other mortality in comparison to those abstaining from statin usage in model 3 (adjusted HR 0.70; 95% CI, 0.51–0.95; adjusted HR, 0.70; 95% CI, 0.50–0.99; adjusted HR, 0.70; 95% CI, 0.53–0.92; respectively). Similar results were presented in sensitivity analyses excluding deaths within two years (eTable 2).

**Table 2:** Comparative hazard ratios (HR) of statin users and nonusers on different causes of mortality across three models—NHANES 1999–2018

Characteristics	Event/All population (weighted)	Model 1		Model 2		Model 3	
		HR (95% CI)	*P*-value	HR (95% CI)	*P*-value	HR (95% CI)	*P*-value
**All-cause mortality**	1471/4,420,636						
No statin		Reference	-	Reference	-	Reference	-
Statin							
Hydrophilic statins		1.06(0.78,1.44)	0.700	1.01(0.76,1.32)	0.960	1.01(0.72,1.41)	0.960
Lipophilic statins (The rest)		0.82(0.71,0.94)	0.010	0.85(0.75,0.97)	0.020	0.77(0.65,0.91)	0.003
**Cancer mortality**	422/1,238,284						
No statin		Reference	-	Reference	-	Reference	-
Statin							
Hydrophilic statins		0.80(0.46,1.39)	0.430	0.72(0.43,1.22)	0.220	0.70(0.37,1.34)	0.280
Lipophilic statins (The rest)		0.71(0.55,0.91)	0.010	0.67(0.52,0.86)	0.001	0.70(0.51,0.95)	0.020
**Cardiovascular mortality**	415/1,247,440						
No statin		Reference	-	Reference	-	Reference	-
Statin							
Hydrophilic statins		1.23(0.72,2.13)	0.450	1.11(0.65,1.90)	0.690	1.08(0.53,2.20)	0.840
Lipophilic statins (The rest)		0.83(0.63,1.09)	0.180	0.87(0.69,1.11)	0.280	0.70(0.50,0.99)	0.040
**Other mortality**	634/1,934,912						
No statin		Reference	-	Reference	-	Reference	-
Statin							
Hydrophilic statins		0.87(0.59,1.28)	0.490	0.85(0.59,1.22)	0.380	0.87(0.57,1.32)	0.500
Lipophilic statins (The rest)		0.74(0.59,0.93)	0.010	0.82(0.65,1.03)	0.090	0.70(0.53,0.92)	0.010

Model 1: Adjusted for age. Model 2: Adjusted for age, sex, and race. Model 3: Adjusted for age, sex, race, education level, body mass index, alcohol use, smoking status, family diabetes mellitus, family cardiovascular disease, diabetes mellitus, cardiovascular disease, dyslipidemia, hypertension and years since the first cancer diagnosis

CI, Confidence interval; HR, Hazard ratio

To further clarify the relationship between statin use and the long-term prognosis of cancer survivors, we compiled the statin use from the onset of cancer to death in ageing cancer population (Table 3). The population taking lipophilic statins after cancer diagnosis showed a decreased risk of mortality in all adjusted models including all-cause mortality (HR, 0.71, *P* < 0.001, in Model 3), cancer-specific mortality (HR, 0.63, *P* = 0.003, in Model 3), cardiovascular mortality (HR, 0.52, *P* < 0.001, in Model 3), and mortality due to other causes (HR, 0.69, *P* = 0.005, in Model 3).

**Table 3:** Comparative hazard ratios (HR) for statin users Versus nonusers regarding mortality from cancer onset to death, across three analytical models—NHANES 1999–2018

Characteristics	Model 1		Model 2		Model 3	
	HR (95% CI)	*P*-value	HR (95% CI)	*P*-value	HR (95% CI)	*P*-value
**All-cause mortality**						
No statin	Reference	-	Reference	-	Reference	-
Statin						
Hydrophilic statins	0.81(0.57,1.16)	0.250	0.74(0.52,1.05)	0.090	0.64(0.44,0.92)	0.020
Lipophilic statins (The rest)	0.76(0.65,0.90)	< 0.001	0.75(0.64,0.88)	< 0.001	0.71(0.60,0.84)	< 0.001
**Cancer mortality**						
No statin	Reference	-	Reference	-	Reference	-
Statin						
Hydrophilic statins	0.73(0.38,1.39)	0.330	0.61(0.33,1.13)	0.120	0.49(0.25,0.96)	0.040
Lipophilic statins (The rest)	0.68(0.52,0.89)	0.004	0.62(0.48,0.81)	< 0.001	0.63(0.46,0.85)	0.003
**Cardiovascular mortality**						
No statin	Reference	-	Reference	-	Reference	-
Statin						
Hydrophilic statins	0.87(0.47,1.62)	0.660	0.66(0.34,1.29)	0.220	0.48(0.23,1.00)	0.050
Lipophilic statins (The rest)	0.70(0.51,0.97)	0.030	0.66(0.49,0.89)	0.010	0.52(0.36,0.74)	< 0.001
**Other mortality**						
No statin	Reference	-	Reference	-	Reference	-
Statin						
Hydrophilic statins	0.61(0.40,0.93)	0.020	0.55(0.35,0.84)	0.010	0.51(0.32,0.83)	0.010
Lipophilic statins (The rest)	0.69(0.53,0.89)	0.010	0.72(0.56,0.92)	0.010	0.69(0.53,0.89)	0.005

Model 1: Adjusted for age. Model 2: Adjusted for age, sex, and race. Model 3: Adjusted for age, sex, race, education level, body mass index, alcohol use, smoking status, family diabetes mellitus, family cardiovascular disease, diabetes mellitus, cardiovascular disease, dyslipidemia, hypertension and years since the first cancer diagnosis

CI, Confidence interval; HR, Hazard ratio

Similar patterns were observed for the aging cancer survivors taking hydrophilic statins. The users of hydrophilic statins showed a reduction in all-cause mortality risk (HR, 0.64, *P* = 0.020) and cancer-specific mortality (HR, 0.49, *P* = 0.040), cardiovascular mortality (HR, 0.48, *P* = 0.050), and mortality due to other causes (HR, 0.51, *P* = 0.010) in the most comprehensive model.

## Discussion

The aging cancer survivors taking statins may exhibited decreased all-cause mortality risk, cancer-cause mortality risk, and also the risk of cardiovascular mortality, and other mortality. Notably, lipophilic statins are significantly associated with lower all-cause and cancer-cause mortality risks among aging cancer survivors.

Previous studies showed statins use significantly diminishes the mortality rate in breast cancer patients [7]. Prior investigations have shown that extended use of statin drugs might lower the risk of developing pancreatic cancer [26], whereas the administration of statins, notably simvastatin, has been linked to a reduced risk of breast cancer relapse [27]. Moreover, a marked reduction in both all-cause and cancer-specific mortality rates has been associated with statin use in aging individuals diagnosed with rectal cancer [28]. In align with their reports, our study extends the benefit of statins use to the general aging cancer survivor demographic. Another interesting finding from our study is that elderly cancer survivors who use statins exhibit lower LDL-C levels compared to those who do not use statins. Guidelines recommend that patients with a moderate (7.5% to < 20%) and high (> 20%) 10-year ASCVD risk score should consider moderate and high-intensity statin therapy, respectively [29]. Compared to the general population, survivors of various adult cancers face an increased medium to long-term risk of cardiovascular diseases (including heart failure, coronary artery disease, arrhythmias, stroke, and venous thromboembolism) [30]. This suggest that the benefits of statin medication may stem from lipid reduction.

Our research highlights the more significant effect of lipophilic statins in preventing mortality within this population, compared with hydrophilic statins. Consistently, earlier studies have emphasized the superiority of lipophilic statins over hydrophilic ones in mitigating complications associated with hepatocellular carcinoma [31]. Furthermore, a meticulous subgroup analysis revealed that this benefit is magnified in male survivors who are above the age of 65, particularly in those grappling with obesity, hypertension, dyslipidemia, and in individuals who are not diabetic. However, we found that both hydrophilic and lipophilic statins are effective in lowering the risk of cancer in aging cancer survivors from the onset of cancer to death, with hydrophilic statins demonstrating superior efficacy.

If statin medications prove to be effective in battling tumor growth, potentially serving as a more strategic and well-tolerated alternative to traditional chemotherapy agents [32], this prospect unveils fresh trajectories for upcoming research endeavors. Subsequent studies can delve deeper into the viable applications of statin drugs, either as standalone agents or in synergy with other medications, in both the prevention and treatment of cancer [27]. These discoveries carry substantial clinical relevance [33], signaling a potentially promising therapeutic pathway in clinical settings. More precisely, the deployment of lipophilic statin medications might establish a targeted modality to mitigate the mortality risk stemming from cardiac events and malignancies [34]. This positive association could signify a groundbreaking shift in the medical management strategies for cancer survivors, facilitating improved survival rates and elevating their life quality [35]. Looking forward, comprehensive investigations are imperative to fully delineate the extent and possible applications of these encouraging findings within the clinical sphere, laying the groundwork for bespoke treatment plans for individuals who have overcome cancer [36]. The intrigue generated by these findings concerning the prospective role of statin drugs in cancer therapy sets a robust platform for forthcoming research initiatives and clinical integrations [37].

### Limitations

Our study presents several notable limitations. Firstly, it is important to recognize that as an observational study, there are inherent limitations in controlling for all potential confounding factors. While we have made efforts to adjust for known confounders in the Models 1–4, there may still be unmeasured or unaccounted variable. Consequently, we cannot offer definitive proof of causation. But we conducted sensitivity analyses excluding deaths within two years, which reduces the possibility of reverse causation. Secondly, the utilization of self-reported data for defining key variables like age, gender, and race introduces a potential bias, as this data might not be entirely accurate or reflective of the actual parameters. Thirdly, our categorization of participants, particularly in relation to statin use, might overshadow more subtle effects of different statins on individuals, thereby not encapsulating the complex realities of patient experiences fully.

## Conclusion

In this study, we identified a significant potential role for statin use, particularly lipophilic statins, in reducing all-cause, cardiovascular, and cancer-specific mortality among aging cancer survivors. Our findings propose the statins may improve the outcomes of aging cancer survivors and provide an insight for a new therapeutic strategy for cancer management.

## Electronic supplementary material

Below is the link to the electronic supplementary material.

Supplementary Material 1

Supplementary Material 2

## Data Availability

No datasets were generated or analysed during the current study.
