# Correction: Association between statin usage and mortality outcomes in aging U.S. cancer survivors: a nationwide cohort study

**DOI:** 10.1007/s40520-024-02900-w

**Published:** 2025-02-27

**Authors:** Shan Ding, Fengling Yang, Pan Lai, Jiang Weiwen, Minze Chen, Yijun Ge, Liting Zhou, Shaozhuang Chen, Jiaqi Zhang, Ye Yanrong

**Affiliations:** 1 Zhongshan Hospital (Xiamen), Fudan University, Xiamen, 361015 China; 2 Longyan First Affiliated Hospital of Fujian Medical University, Longyan, 364000 China; 3 Peking University First Hospital, Beijing, 100034 China; 4 Zhongshan Hospital, Fudan University, Shanghai, 200032 China; 5 Faculty of Health and Environmental Sciences, Auckland University of Technology, Auckland, 0627 New Zealand; 6 Shenzhen Campus of Sun Yat-Sen University, Shenzhen, 518107 China

**Correction: Aging Clinical and Experimental Research (2024) 36:200**

10.1007/s40520-024-02851-2

In this article, the author's affiliation details have been incorrectly labeled as

Weiwen Jiang^1^ · Minze Chen^4^ · Yijun Ge^5^ · Yanrong Ye^1^

and should have been as

Jiang Weiwen^1,4^, · Minze Chen^5^ · Yijun Ge^6^ · Ye Yanrong^1,4^

The original article has been corrected.

