# Supplementary material for: Association between statin usage and mortality outcomes in aging U.S. cancer survivors: a nationwide cohort study
Source: Aging Clin Exp Res. 2024 Oct 5;36(1):200. doi: 10.1007/s40520-024-02851-2 (PMC11458640; doi:10.1007/s40520-024-02851-2)
Supplement: Supplementary file 1 — Supplementary Material 1 [file 40520_2024_2851_MOESM1_ESM.docx]

| **Variable** | **Total** | **Female** | **Male** |
| --- | --- | --- | --- |
| **Cancer style** |  |  |  |
| **Bladder** | 116 | 28 | 88 |
| **Blood** | 8 | 2 | 5 |
| **Bone** | 19 | 5 | 14 |
| **Brain** | 8 | 1 | 7 |
| **Breast** | 475 | 474 | 1 |
| **Cervix (cervical)** | 46 | 46 | 0 |
| **Colon** | 274 | 133 | 141 |
| **Esophagus (esophageal)** | 20 | 5 | 15 |
| **Gallbladder** | 1 | 1 | 0 |
| **Kidney** | 67 | 21 | 46 |
| **Larynx/ windpipe** | 17 | 1 | 16 |
| **Leukemia** | 34 | 12 | 22 |
| **Liver** | 21 | 11 | 10 |
| **Lung** | 102 | 38 | 64 |
| **Lymphoma/ Hodgkin's disease** | 25 | 17 | 8 |
| **Lymphoma/Hodgkin's disease** | 22 | 10 | 12 |
| **Melanoma** | 195 | 79 | 116 |
| **Mouth/tongue/lip** | 18 | 3 | 15 |
| **Other** | 142 | 74 | 68 |
| **Ovary (ovarian)** | 54 | 54 | 0 |
| **Pancreas (pancreatic)** | 7 | 3 | 4 |
| **Prostate** | 649 | 0 | 649 |
| **Rectum (rectal)** | 23 | 10 | 13 |
| **Skin (don't know what kind)** | 298 | 123 | 175 |
| **Skin (non-melanoma)** | 520 | 210 | 310 |
| **Soft tissue (muscle or fat)** | 6 | 2 | 4 |
| **Stomach** | 25 | 12 | 13 |
| **Testis (testicular)** | 9 | 0 | 9 |
| **Thyroid** | 38 | 30 | 8 |
| **Uterus (uterine)** | 101 | 101 | 0 |

**eTable 1. Numbers of Cancer Survivors by Cancer Type and Sex, NHANES 1999-2018**
