# Supplementary material for: Association between statin usage and mortality outcomes in aging U.S. cancer survivors: a nationwide cohort study
Source: Aging Clin Exp Res. 2024 Oct 5;36(1):200. doi: 10.1007/s40520-024-02851-2 (PMC11458640; doi:10.1007/s40520-024-02851-2)
Supplement: Supplementary file 2 — Supplementary Material 2 [file 40520_2024_2851_MOESM2_ESM.docx]

**eTable 2 Comparative Hazard Ratios (HR) of Statin Users and Nonusers on Different Causes of Mortality Across Three Models** **excluding deaths within two years—NHAMES 1999–2018.**

| **Characteristics** | **Model 1** | |  | **Model 2** | |  | **Model 3** | |
| --- | --- | --- | --- | --- | --- | --- | --- | --- |
|  | **HR (95% CI)** | **P-value** |  | **HR (95% CI)** | **P-value** |  | **HR (95% CI)** | **P-value** |
| **All-cause mortality** |  | |  |  | |  |  | |
| No statin | Reference | - |  | Reference | - |  | Reference | - |
| Statin |  |  |  |  |  |  |  |  |
| Hydrophilic statins | 1.12(0.81,1.55) | 0.500 |  | 1.05(0.78,1.41) | 0.760 |  | 1.13(0.79,1.62) | 0.500 |
| Lipophilic statins (The rest) | 0.82(0.70,0.97) | 0.020 |  | 0.87(0.75,1.00) | 0.060 |  | 0.78(0.65,0.94) | 0.010 |
| **Cancer mortality** |  |  |  |  |  |  |  |  |
| No statin | Reference | - |  | Reference | - |  | Reference | - |
| Statin |  |  |  |  |  |  |  |  |
| Hydrophilic statins | 0.81(0.45,1.44) | 0.470 |  | 0.72(0.41,1.27) | 0.260 |  | 0.83(0.43,1.62) | 0.590 |
| Lipophilic statins (The rest) | 0.66(0.49,0.91) | 0.010 |  | 0.63(0.46,0.85) | 0.003 |  | 0.63(0.44,0.91) | 0.010 |
| **Cardiovascular mortality** |  | |  |  | |  |  | |
| No statin | Reference | - |  | Reference | - |  | Reference | - |
| Statin |  |  |  |  |  |  |  |  |
| Hydrophilic statins | 1.38(0.75,2.56) | 0.300 |  | 1.23(0.67,2.26) | 0.500 |  | 1.23(0.57,2.68) | 0.600 |
| Lipophilic statins (The rest) | 0.92(0.69,1.22) | 0.560 |  | 0.98(0.76,1.27) | 0.870 |  | 0.77(0.55,1.09) | 0.140 |
| **Other mortality** |  | |  |  | |  |  | |
| No statin | Reference | - |  | Reference | - |  | Reference | - |
| Statin |  |  |  |  |  |  |  |  |
| Hydrophilic statins | 0.90(0.59,1.38) | 0.630 |  | 0.87(0.59,1.29) | 0.490 |  | 0.93(0.59,1.46) | 0.750 |
| Lipophilic statins (The rest) | 0.73(0.57,0.93) | 0.010 |  | 0.81(0.64,1.03) | 0.090 |  | 0.72(0.54,0.96) | 0.020 |

*Model 1: Adjusted for age. Model 2: Adjusted for age, sex, and race. Model 3: Adjusted for age, sex, race, education level, body mass index, alcohol use, smoking status, family diabetes mellitus, family cardiovascular disease, diabetes mellitus, cardiovascular disease, dyslipidemia, hypertension* *and years since the first cancer diagnosis.*

*CI, Confidence interval; HR, Hazard ratio.*
